# Genome Integrity - a new open access journal

**DOI:** 10.1186/2041-9414-1-1

**Published:** 2010-05-26

**Authors:** Razqallah Hakem, M Prakash Hande, John Petrini, Predrag Slijepcevic

**Affiliations:** 1 University of Toronto, Canada; 2 National University of Singapore, Singapore; 3 Memorial Sloan-Kettering, Cancer Center, New York, USA; 4 Brunel University, UK

## 

The field of DNA damage response and the associated cellular processes behind genome stability maintenance constitute one of the hottest research areas in modern biomedical sciences. A quick search on PubMed using the key word "DNA damage" results in approximately 400 articles which were published in January 2010 [1]. Similarly, ~300 articles that contain key words "DNA repair" in the abstract were published in the same period. The large majority of these articles are published through the conventional publishing model which implies that: (i) readers only have access to articles through subscriptions and (ii) there is a gap of several weeks between the acceptance of articles and access to them online or in print.

By launching *Genome Integrity*, the first open access journal dedicated to the field of DNA damage response and associated processes, we aim to provide interested scientists with the journal that enables (i) immediate online access to articles as soon as they are accepted for publication and (ii) free and universal online access resulting in dissemination to the widest possible audience. We believe that the current lack of opportunities for immediate and free dissemination of articles focusing on the above area of research will make *Genome Integrity *a viable and competitive journal. We would like to note that *Genome Integrity *articles will be archived in PubMed [1] and all freely accessible full-text repositories. This complies with the policies of a number of funding bodies including the Wellcome Trust, NIH and Howard Hughes Medical Institute [2-5].

The scope of *Genome Integrity *is wide and ambitious. We aim to attract articles focusing on all aspects of DNA damage response mechanisms, including mechanisms of DNA damage induction, sensing, signalling and repair, cell cycle check-point control, telomere maintenance and control of apoptosis. The journal also welcomes submissions which focus on mechanisms of chromosome stability maintenance and the effects of genotoxic stress on this stability. A growing area of research within the field is understanding DNA damage processing in the context of interphase nucleus chromatin and the journal certainly aims to attract authors interested in the mechanisms underlying these processes. *Genome Integrity *also intends to encourage publications from authors interested in exploring the effects of normal and pathological DNA damage responses on tissue homeostasis, cellular and organismal ageing and tumorigenesis in humans and in animal models. In brief, *Genome Integrity *will publish articles exploring fundamental, as well as translational, aspects of all processes behind DNA damage response, genome and chromosome stability maintenance.

To ensure the scientific quality of the journal we will use a rigorous and transparent peer-review process. All articles will be reviewed by at least two experts who are expected to provide an independent assessment of suitability of articles for publication in *Genome Integrity*. In order to make *Genome Integrity *even more competitive we will aim to make editorial decisions within the shortest possible period which, when all practicalities are taken into account, should be 6 weeks from the date the article was submitted through the journal submission system. We will select reviewers, based on their expertise, either from the *Genome Integrity *Editorial Board currently consisting of ~50 leading scientists in the field [6], or from the wider scientific community. Editors will regularly consult with the Editorial Board members to ensure a smooth progression from the initial launch to the journal's recognised status as a fully established publishing platform on all aspects of DNA damage response mechanisms.

We would also like to highlight some advantages of the open access publishing model in this Editorial. First, open access journals have the potential to reach a much larger set of readers than any subscription-based journal, in print and online [7]. Second, some studies have suggested a correlation between open access, higher downloads and higher citations, leading to a higher Impact Factor [8,9]. Third, authors hold copyright for their work and grant anyone the right to reproduce and disseminate the article, provided that it is correctly cited. Finally, financial or grant status of the researcher will not influence his/her ability to access articles, this is because investigators or the public who are interested in this field of science in resource-poor countries will be able to read the same material as their counterparts in resource-rich countries [10].

We are very excited about the launch of *Genome Integrity*. We strongly believe that this new journal will significantly contribute to understanding of DNA damage response processes and look forward to receiving manuscripts from many colleagues.
